# Experiences of patient organizations’ involvement in medicine appraisal and reimbursement processes in Finland – a qualitative study

**DOI:** 10.1017/S0266462324000229

**Published:** 2024-07-02

**Authors:** Mirjami Tran Minh, Marja Airaksinen, Tuuli Lahti

**Affiliations:** 1 Faculty of Medicine, University of Helsinki, Helsinki, Finland; 2 Faculty of Pharmacy, University of Helsinki, Helsinki, Finland; 3 Health and Well-being, Master School, Turku University of Applied Sciences, Turku, Finland

**Keywords:** patient involvement, patient input, health technology assessment, HTA, health policy

## Abstract

**Background:**

This study investigated how patient representatives have experienced their involvement in medicines appraisal and reimbursement processes with the Council for Choices in Health Care in Finland (COHERE) and the Pharmaceuticals Pricing Board (PPB) and how authorities perceive the role of patient organizations’ input.

**Methods:**

Semi-structured thematic individual and pair interviews were conducted in 2021 with representatives (n = 14) of patient organizations and government officials (n = 7) of the Ministry of Social Affairs and Health. The interview data were analyzed using qualitative content analysis.

**Results:**

Patient representatives expressed their appreciation for the PPB and the COHERE in creating consultation processes and systematic models that support involvement. However, there were many challenges: patient representatives were uncertain about how their submissions were utilized in official processes and whether their opinions had any significance in decision-making. Patients or patient organizations lack representation in appraisal and decision-making bodies, and patient representatives felt that decision-making lacked transparency. The importance of patient involvement was highlighted by the authorities, but they also emphasized that the patient organizations’ contributions were complementary to the other materials. Submissions regarding the medications used to treat rare diseases and those with limited research evidence were considered particularly valuable. However, the submissions may not necessarily have a direct impact on decisions.

**Conclusions:**

The interviews provided relevant input for the development of involvement processes at the PPB and COHERE. The interviews confirmed the need for increased transparency in the medicines assessment, appraisal, and decision-making procedures in Finland.

## Background

The involvement of patient representatives is an increasingly important part of the assessment of healthcare technologies (HTA) and decision-making concerning the recommendations or reimbursement of medicines (1–8). Patient and public involvement (PPI) is based on the understanding that patients and the public are stakeholders who are equal to clinicians and other experts in the processes that support HTA-informed decision making and in priority setting in general (2;9). Patients are directly affected by HTA decisions – they are key stakeholders and have a democratic right to be involved (9). It is also widely acknowledged that the experiential knowledge patients hold can contribute to the quality and relevance of HTA (6;7;9). There are considerable variations in scope, methodology, and practices in conducting PPI, and involvement can be seen as a continuum ranging from information dissemination and consultations led by authorities to collaboration and equal partnership (4;10–13).

In Finland, the opportunities for PPI have increased, albeit slowly compared to pioneers such as the National Institute for Health and Clinical Excellence in England and the Canadian Agency for Drugs and Technologies in Health (14;15). In 2016, the Pharmaceuticals Pricing Board (PPB), responsible for national price and reimbursement decisions in Finland, opened the possibility for patient organizations to provide written submissions on the therapeutic value of medicines from a patient perspective (16). In the same year, the Council for Choices in Health Care in Finland (COHERE), responsible for issuing recommendations on services that should be included in the range of public health services in Finland, opened its draft recommendations for public comment through the Ministry of Justice’s online platform called *Ota kantaa* (in English “Have Your Say”) (17).

According to a previous Finnish study, patient organizations have provided written submissions to the PPB and COHERE, but to a limited extent (18;19). From 2017 to 2021, the PPB has received 17 to 31 submissions annually for price and reimbursement applications from patient organizations. During the same period, the annual number of submissions to the COHERE’s draft recommendations varied from 0 to 26 (19).

### Aims of this study

The aim of this study was to review patient representatives’ experiences of involvement in the medicine appraisal and reimbursement processes in Finland. The specific objective of this study was to discover how patient representatives have experienced their involvement in medicine-related processes with the PPB and COHERE. We also investigated how authorities perceive the role of patient organizations’ input in the appraisal and decision-making processes of the PPB and the COHERE.

## Methods

### Context of the study – Finnish medicine appraisal and reimbursement system

In Finland, multiple organizations are involved in the national assessment, appraisal, and decision-making of publicly funded medicines. In this study, we focused on the PPB, which makes decisions regarding the price and reimbursement for outpatient care medicines, and the COHERE, which issues recommendations on hospital (inpatient care) medicines. The tasks of these two organizations and the opportunities for PPI are presented in Table 1.

**Table 1.** Tasks of the Pharmaceuticals Pricing Board (PPB) and the Council for Choices in Health Care in Finland (COHERE) and opportunities for patient involvement

Organization	PPB	COHERE
Task	Makes decisions on the reimbursement and reasonable wholesale prices of outpatient medicines (prescription medicines purchased by outpatients from the community pharmacy) and clinical nutritional products based on the price and reimbursement applications submitted by the marketing authorization holder (20). There are different types of applications, such as those for new active substances, for the extension of reimbursement status, and for renewals (21).	Issues recommendations on services that should be included in the range of public health services in Finland, including hospital–only medicines. The COHERE’s pharmaceutical division prepares the recommendations for the medicines administered in a hospital setting together with the COHERE’s secretariat, practically evaluating all hospital–only medicines assessed by the Finnish Medicines Agency’s (Fimea) HTA team (22).
Legislation	Health Insurance Act (1224/2004) (20)	Health Care Act (Sections 7a and 78a) (23)
Implementation of decision/recommendation	Decisions are implemented by the National Insurance Institution (Kela) and are applied nationwide.	Recommendations are not binding but they are generally followed by Finnish hospitals. In case of conditional recommendation, national price negotiations are conducted by the HUS Helsinki University Hospital pharmacy.
Opportunities for patient involvement	Possibility to send written submission as an email attachment to the secretariat since 2016. The patient organizations have 30 weekdays to send a submission to the PPB.	Possibility to give written submission through *Ota kantaa* webservice since 2016; possibility to comment on the topic while draft recommendation is being prepared (since 2022); occasional meetings with patient representatives. The consultation period is approximately 3 weeks.
Information provided for patient organizations	The PPB publishes a list of the price and reimbursement applications submitted by the pharmaceutical companies during the previous month. The list includes name of the medicinal product and type of application (new active substance, generic product, renewal application). The list is available on the PPB’s website and is sent by e–mail to the patient organizations that have signed up for the PPB’s mailing list. The PPB has published guidelines for patient organizations’ submissions in Finnish that provide instructions on the content and structure of the submissions (24). The guidelines call for the inclusion, in particular, of perspectives on the need for the medicinal product and its relationship with other drug– or drug–free treatments in accordance with the current treatment practice, the users’ experiences of the medicinal product and its effects on the ability to function, coping with everyday life and the quality of life, as well as on the factors that may limit the use of the medicinal product. The PPB instructs the patient organizations to declare any interests related to the matter or the application.	The COHERE informs the public about upcoming and ongoing evaluations as well as the draft recommendations available for comment on its website and in the newsletter to which the public subscribes on the COHERE’s website. When submitting the comment, COHERE requests to inform from which perspective the comment is given: from the perspective of a patient, customer, or carer, patient organization, healthcare professional, service provider, and so forth. The purpose of public consultation and general instructions for commenting on COHERE’s draft recommendations can be found in its handbook (25). The purpose is primarily to ensure that all relevant material is taken into account, that the recommendation is based on accurate information, that different aspects related to the matter are sufficiently considered and that the recommendation is understandable in terms of language.
Feedback for patient organizations	The PPB does not publish any feedback on submissions, but the decisions are published on the website after each PPB meeting.	On most occasions, COHERE prepares a summary of the submissions and feedback that is included in the final recommendation documents, which are available online.

### Study setting and recruitment of interviewees

This study was performed using qualitative methods, as its aim was to investigate and increase the understanding of the views and experiences of both the patient representatives and authorities. No previous research on this topic has been performed in Finland.

The research data consist of semi-structured interviews conducted with patient representatives and authorities. The interviewees were selected on a discretionary basis; the selection criteria for interviewees required that they were either an employee or a board member of a patient organization or, in the case of authorities, that the work of an individual government official was related to the activities of either the PPB or the COHERE.

In this study, we referred to patient organizations’ employees or trustees as *patient representatives,* which is a common term to use for “A person or organization who/that is actively involved with others and presents the perspectives and concerns of a group of patients” (26).

Stakeholders were selected using purposive sampling to ensure the coverage of all relevant perspectives (27). The recruitment of patient representatives was carried out through the Network of Patient and Public Health Organizations, which is coordinated by the SOSTE Finnish Federation for Social Affairs and Health. The research project was presented at the network meeting and on the network email list. In 2021, the network had approximately 40 member organizations. Direct e-mails were also sent to the staff of 11 selected patient organizations representing the patient groups for which new medicines had been authorized according to the EMA Human Medicines Highlights (28). The interviews were conducted with the goal of gathering representative perspectives from the patient community; thus, direct recruitment was carried out to invite patient organizations of different sizes and backgrounds, including established large patient organizations and newer volunteer-run organizations. The authorities were invited to participate in the interviews by sending emails to the secretariats of the PPB and the COHERE. The invited organizations autonomously designated the individual(s) to take part in the interview.

### Interview guide

The semi-structured interview guide was developed in collaboration with the patient organizations and tested with an employee of the patient organization before the actual interviews began. Based on the pilot interview, minor changes were made to the interview guide, simplifying two questions, and adding two clarifying questions. The pilot interview was not included in the data analysis. Although the themes were the same, the questions were different for the patient representatives and the authorities to reflect the different roles and responsibilities of the parties. The full interview guide consisted of multiple questions covering the following themes: the processes of the PPB and the COHERE, the experiences of the involvement and significance of the submissions, the information provided by the authorities, and the possible needs for support and education (Supplementary material S1). In this study, we concentrated on the questions related to the experiences of the involvement, role, and significance of the patient organizations’ input as perceived by both the authorities and patient representatives (Table 2).

**Table 2.** The questions for patient representatives and authorities included in this study

The questions for patient representatives, translated from the original Finnish language interview guide to English:
What has been your experience of providing submissions and/or collaborating with the PPB and COHERE? How is the involvement of patient organizations implemented in the official processes related to the reimbursement of medicines (PPB) and the preparation of medicine–related recommendations (COHERE)? Do you see that the submissions you have given have been taken into account in the PPB’s or the COHERE’s decision making? How is information on submissions returned to the patient organization?
**The questions for authorities, translated from the original Finnish language interview guide to English:**
What is your experience of the involvement of patient organizations in the processes of PPB and COHERE? What role do you see for patient organizations and patient organization submissions in the operations of PPB or COHERE? How are patient organization submissions taken into account in the PPB’s or the COHERE’s decision making? Are the submissions made so that they are useful in the PPBs or COHERE’s operations? How could they be developed in terms of content?

### Data collection

Prior to the interview, the interviewees received an email with an information sheet about the study, an informed consent form and an interview guide, and a data protection notice for scientific research. The interviewees provided their consent to participate in the interview survey and to use their personal data. The participants also gave informed consent to the recording of the interview.

All interviews were conducted by the first author MTM, with qualitative research training as part of her doctoral studies. The interviews were conducted via Teams video calls and recorded separately as audio files. At the beginning of each interview, the participants were informed orally about the study’s objectives and background and the voluntary nature and confidentiality of their participation.

The interviews were conducted using the interview guide as a framework, but the order in which the themes were discussed varied depending on the natural progression of the conversation. The interview guide was used to ensure that all the participants addressed the themes relevant to the research questions (29). An external company transcribed the audio recordings, and the first author of the article checked the transcriptions and removed any identifying information from them.

### Data analysis

The first author of this article analyzed the interview data using inductive content analysis with Atlas.ti (version 22). The transcribed material was read several times, encoded, and structured by category (subcategory, upper category, main category). The data analysis was carried out in stages. In the first stage, the researchers familiarized themselves with the data as a whole. In the second stage, the analysis of the transcribed data was carried out deductively, with the research questions (Table 2) guiding the classification of the data: text passages corresponding to or related to these questions were searched in the data, and these were marked with verbal codes in the Atlas.ti program (30). In the third stage, the encoded text passages were analyzed inductively, that is, data-driven and grouped into categories according to the principles of content analysis. The quotations in this article were selected to represent the themes that were often repeated in the interviews. To protect the identity of the interviewees, the citations are not specified in detail, only the interviewee group (patient representative or government official). The citations were translated from Finnish to English by the authors and were checked by a native English speaker. The comments from the authors are in parentheses, for example, when a name has been removed from the text or an explanation is needed.

### Research ethics

The research followed the good scientific practices and ethical principles drawn up by the Finnish National Board on Research Integrity in research related to the human sciences (31). The study did not include nonmedical research designs related to the human sciences that require a preliminary assessment by an ethics committee (31;32).

## Results

### Participants

The interviews were conducted between June and November 2021. A total of 21 individuals participated in the interviews, including 14 patient representatives from 10 patient organizations and 7 government officials (Supplementary material S2). The number of interviewees was dependent on the positive response to the interview invitations sent to the patient organizations and secretariats of the PPB and the COHERE. Of these interviews, 4 were conducted in pairs, and 13 were conducted individually. All 7 interviews with government officials were conducted as individual interviews. To protect the privacy of the interviewees, the background information of the interviewees is not detailed in the article.

The interview duration varied from 19 to 65 minutes, with an average duration of 40 minutes. The total length of the audio files was 681 minutes (11 hours and 21 minutes) (Supplementary material S2).

### Experiences of involvement

Several of the patient representatives mentioned that the formal processes for the submissions to the PPB and the COHERE work well (Table 3). The patient representatives were given the opportunity to provide written submissions, and clear structures were established for this purpose.

**Table 3.** Patient representatives’ experiences of involvement in the PPB and COHERE consultative processes

Description	Citation from interview
The formal process is considered fine	*At the moment, in my opinion, it’s in a pretty good state with a well–established structure. It’s not erratic but has a certain procedure which is adhered to.* *In itself, the opportunity to provide that submission already enables participation. But of course, how it is done within the patient organization, how that submission is formed, there should be some expertise and prudence on the part of the organization.*
Feeling of tokenism	*There is a general feeling that we are only kept involved because this will keep us satisfied at some level.*
Uncertainty about how the authority utilizes the submissions	*We don’t know if our perspectives have been seen, heard, or considered, and there is no real dialogue happening.*
Lack of transparency	*[I] should have been present at the meeting myself to understand what has been going on. And in that regard, this kind of openness and transparency, in my opinion, is not being implemented.* *In my opinion, a certain level of transparency has been lacking in this.*
Weak sense of participation and involvement	…*and there are no follow–up questions, so the sense of participation is, let’s say, somewhat weak.* *It doesn’t really happen, it’s a one–way process.*

However, a few interviewees felt that participation was superficial: the opportunities to provide submissions were created to keep the patient organizations satisfied. Despite these feelings, the patient representatives acknowledged that the authorities are doing their best within the limited resources and powers allocated to them.

### Uncertainty of how authorities use the submissions

Despite the advances in the formal processes, many interviewees mentioned that the patient representatives do not know how authorities utilize statements and whether they have any significance in the appraisal and decision-making processes (Table 3).

Although they hope and would like to believe that their submissions have significance and impact, there was a strong feeling of uncertainty, as they do not receive any feedback on individual submissions. Thus, the submission process appeared to the patient representatives to be a one-way process. The patient representatives monitor the reimbursement decisions on the PPB’s website, follow the COHERE newsletter and website for final recommendations, and reflect on the decisions in relation to their submission. Regarding the COHERE, some patient representatives mentioned that the final recommendations were less restrictive than the draft recommendations and, based on that, the submissions might have had an impact. However, several patient representatives acknowledged that their submissions were a small part of the process and that there were other aspects that were more strongly emphasized.

Many interviewees expressed a desire for feedback on the submissions and their significance so they could pass the feedback to the members of their organization who have contributed to the submissions and shared personal experiences of their illnesses.

The uncertainty regarding how authorities handle the submissions was associated with a lack of transparency. There are no patient or civil society representatives present in the appraisal or decision-making bodies or the advisory boards of the PPB or the COHERE, which was brought up by a couple of the interviewees.

As a result of these uncertainties and the lack of transparency and feedback, the overall experience of participation was considered weak by several patient representatives (Table 3).

### Role and value of patient organizations’ input as perceived by authorities

The authorities emphasized the value of patient input but also indicated that the patient organizations’ submissions are complementary to the large application dossiers prepared by the pharmaceutical companies in the PPB and/or the assessment reports compiled by the national medicines’ authority FIMEA and the COHERE and that decisions are always made based on an overall evaluation and legislation (especially in the case of PPB).

Several of the interviewees did not recall a situation in which patient input would have completely reversed the outcome or decision; however, the input can support or strengthen the final decision and complement the input from other stakeholders, such as medical doctors.

The perceived significance of the submissions as experienced by the authorities is presented in Table 4.

**Table 4.** Significance of submissions from the perspective of authorities

Description	Citation from interview
Decisions are based on an overall assessment, and the submissions from patient organizations are one part of the whole.	*The deliberation carried out by the Pharmaceuticals Pricing Board is always a comprehensive assessment. It consists of many small pieces.* *Therefore, in a way, it’s important to always consider that the work of the PPB involves an overall evaluation. As boring as it may sound, it is based on the Health Insurance Act.*
Submissions are a good addition to support and strengthen the authorities’ decision–making process.	*Bringing the patient perspective into the discussion is a valuable addition, particularly to the statements of treating physicians and the knowledge obtained from research literature, for example.* *In decision–making processes, I see it more as a reinforcement of the committee’s perspective, especially when there is uncertainty or deliberation regarding a particular matter.*
Submissions are taken into account, but they do not significantly alter the end result.	*Those submissions are always taken into consideration, but their effect on the outcome varies, and their impact is not necessarily significant.* *Rarely does anyone say anything at that stage that completely flips the matter around in the opposite direction.* *I don’t recall any case where the patient organization submission would have completely reversed the outcome.*

### Usefulness of submissions from the perspective of authorities

#### Types and content of submissions that are found useful

The content of the submissions varied, and the different types of submissions had varying values for the authorities. Several of the authorities stated that submissions are particularly useful when dealing with rare diseases or disease subgroups on which there are limited research data (Table 5). In such cases, submissions help authorities to better understand the disease and its impact on patients’ lives.

**Table 5.** The usefulness of patient organization submissions as perceived by authorities

Category	Description	Citation from interview
Content of submissions that are found useful	Understanding the overall treatment landscape including possible problems and unmet needs. Rare diseases and new treatments with limited research evidence Patient perspective on adverse/side effects. Use of meaningful and patient relevant measures.	*I have found them useful when they describe the patient’s perspective, for example, existing treatment options and the overall treatment landscape from the patient’s point of view. It gives an insight into what life is like for the patient with that illness, highlighting the problem areas and whether this new treatment could potentially offer a solution or relief.* *The areas where these submissions are most needed are those for rare diseases and new medications that have a small user base, likely including the most severely ill individuals.* *Where we would welcome the patient perspective is in how they assess the harm and side effects.* *What is truly meaningful for families is the relevance of the measures used in healthcare to their everyday life.*
Content of submissions not found useful	Submissions for renewal applications (to PPB). Mere wish for reimbursement or positive recommendation. Repeating results of clinical trials and treatment guidelines in the submissions. Submission prepared by one person, no wider patient perspective.	*Regarding renewal applications, I don’t really see it as necessary to repeatedly state that reimbursement should be maintained. Often, reimbursement remains in place, and if there are any changes, such as patents expiring or price pressures, the submission from a patient organization doesn’t really help in that regard.* *Then, if the patient organization simply wishes in their statement that the preparation would be reimbursed, it does not truly provide additional information that could be of any particular benefit.* *Then, again, those types of submissions where the patient’s perspective is forgotten. The efficacy of the drug is evaluated through clinical trials and the Current Care Guidelines are familiar to us, we will look at them anyway. There are some submissions that do not add to what we are already looking at in that assessment.* *Sometimes you can tell that is just written by someone [one person] from the patient organization.*

The submissions were considered to be useful and enlightening when they described the existing treatment options and the overall treatment context from the patient’s perspective: what the patient’s life is like with the illness, what the problematic aspects are and whether this new treatment could bring any solutions or relief regarding those issues. The perspectives on how patients evaluate the harms and side effects of drugs were also considered helpful. In short, the authorities expressed the desire for the submissions for reimbursement applications to relate to the topics that are listed in the PPB instructions for patient organizations.

In the case of COHERE, the patient perspectives regarding the relevance of the outcome measures used in the scientific studies and healthcare context were seen as important when assessing the impact of illness on the patients and their carers. Regarding the COHERE’s recommendations, the authorities emphasized that the main goals of consultation are to ensure that all the relevant material is considered, that the recommendation is based on accurate information, and that all the relevant aspects are sufficiently considered. In addition, it is important that the recommendation is written in a comprehensible manner that facilitates the implementation of the recommendations in healthcare.

The authorities recognized that the personal experiences shared in the submissions may not be objective as such. However, they expressed the desire that the submissions were balanced, not entirely focused on the possible benefits of the new drug but also considering the possible challenges and side effects.

#### Types and content of submissions that are found less useful

Multiple officials stated that, in the case of PPB, it may not be necessary to provide statements for renewal reimbursement applications, because the focus of such applications is primarily on the price, assuming that the therapeutic value of the medication has been deemed sufficient. In situations where both patient organizations and authorities have limited resources, providing submissions for renewal applications can even be considered a waste of resources.

Additionally, if a patient organization primarily expresses a wish for reimbursement or a positive recommendation of a medicine in their submission, it might not provide any additional information for the authorities that could be of a specific benefit. Furthermore, submissions were found to be useless when the patient perspective was absent, and the submissions focused primarily on reporting the scientific findings or treatment recommendations that the authorities were previously aware of (Table 5).

## Discussion

Significant progress has been made in incorporating patients and patient organizations into the participatory processes of PPB and COHERE over the past decade. However, despite these advancements, numerous challenges persist. Patient representatives raised concerns about the meaningful integration of their opinions into decision-making processes, and the involvement process was seen as tokenistic.

The uncertainty of the impact of patient group input and the lack of feedback on submissions have also been acknowledged in other studies (15;33). Additionally, the challenges in integrating patient input into assessment, appraisal, and coverage decisions have been acknowledged in previous studies (15;34).

The authorities acknowledged the significance of patient involvement while also stressing that contributions from patient organizations were supplementary to other materials. The authorities most value submissions that deal with rare diseases and medicines with limited scientific evidence. The patient organizations’ input helps to understand the impact of a condition and treatment on the lives of the patient and carers and provides context for other evidence, in some cases providing reassurance for the decisions of the authorities. The findings of this study are consistent with those of previous studies in the UK and Canada (8;15;35;36). However, the challenge with submissions concerning treatments for rare diseases is that patient organizations face difficulties in finding direct patient experiences with these treatments, which have often received marketing authorization with accelerated approval based on limited clinical evidence (37). As stated by the interviewed authorities, in these cases, it is valuable to share information on the current treatments, the patients’ life experiences with the condition, and its practical burden from the patient’s perspective.

The uncertainty regarding how and whether the authorities use the submissions left the patient representatives with a feeling of tokenism. This was expressed by a couple of patient representatives as a feeling that they are only kept involved to keep the patient organizations satisfied. In Sherry Arnstein’s widely used ladders of participation (38), consultation does indeed fall within the category of tokenism. Arnstein noted that “inviting citizens’ opinions, such as informing them, can be a legitimate step toward their full participation.” Nonetheless, if consultation processes are not accompanied by other forms of participation, this level of the ladder remains inadequate because there is no guarantee that citizen concerns and ideas will be integrated (38). Arnstein’s ladders have been criticized for failing to engage with the complexity and nuances of PPI and for focusing only on outcomes, rather than processes, of involvement (39). In this context, the International Association for Public Participation’s (IAP2) Spectrum of Public Participation emerges as a potentially more relevant framework (40). IAP2’s Spectrum posits that consultation includes a commitment to “keep you informed, listen to and acknowledge concerns and aspirations, and provide feedback on how public input influenced the decision.” However, the consultation models employed by PPB and COHERE, as indicated by the interview findings, do not seem to embody these principles effectively. Another question is whether written consultation is an optimal approach to obtain the information authorities are expecting.

In Finland, patient organizations do not have direct representation on decision-making or appraisal committees or boards, which consist of officials and experts, including pharmacists, medical doctors, lawyers, and health economists. Having direct representation would increase the transparency and legitimacy of the appraisal and decision-making and alleviate tokenism (15;33;41). Direct representation throughout the process has been considered especially important in the assessment and appraisal of orphan medicinal products (37). Nonetheless, according to Sandman et al. (42), the inclusion of a solitary patient representative or a limited number of representatives in a decision-making body does not ensure the acquisition of necessary input. Conversely, there exists a potential bias toward the particular diagnosis or circumstances of the patient representative, and the representative’s viewpoint can be influenced by their individual experiences. However, this potential bias does apply also to other members of the board including healthcare professionals. As an alternative to direct representation, the decision-making process could be rendered more transparent, for instance, by documenting the deliberations on the matter or even by making the deliberations open to the public (42).

In addition to direct representation and increasing transparency in decision making, providing sufficient reporting and feedback on patient organizations’ submissions plays a key role in building common trust and improving the processes and impacts of submissions (7). The importance of feedback as well as clearly articulated purpose and goals for meaningful PPI in HTA and HTA-informed decision-making has been highlighted in several studies and standards (5;7;10;12;13;40;43).

### Policy implications

To enhance dialogue and to improve transparency, it is recommended for authorities to provide feedback on patient organizations’ submissions and their significance in the appraisal and decision-making processes (7;40). The COHERE produces a summary document of the submissions for most of the recommendations in which they mention the amendments that have been made based on the submissions. However, this summary is not available for all recommendations. The PPB does not publish any feedback on submissions; however, the decisions are published on the website after each PPB meeting. Detailed feedback for each submission would be ideal, but even an annual report summarizing the submissions and their significance in supporting the decision making could improve the transparency, impact, and quality of the submissions, as well as the involvement practices, as proposed by earlier studies (41). Reporting would also assist in evaluating and monitoring the impact of involvement activities, which has been recognized as a major gap in implementing PPI (8;13;44;45). Guidelines aimed at patient organizations should better consider what authorities expect from submissions and when statements do or do not contribute to the appraisal or decision-making process. Additionally, clearly stated goals for involvement activities are needed.

Based on the Canadian and UK examples, direct patient input through participation in meetings appeared to be more influential than written patient group submissions. Including patient representation in the deliberative processes has been shown to increase the impact of involvement (8;33;41;46). In the Finnish context, this could include patient representation in the PPB’s and COHERE’s expert groups or decision-making bodies, which could further increase the transparency and legitimacy of operations. Training for both the authorities and patient representatives is required to improve the processes (7;13).

In the European regulatory environment for medicines, significant changes are currently taking place. The EU’s new Regulation 2021/2282 on Health Technology Assessment (HTAR) will be applicable from January 2025 (47). The HTAR will have an impact on the national assessment and appraisal processes and the operations of the PPB and COHERE. The subcommittee for the steering and funding of pharmacotherapy, appointed by the intersectoral coordination group nominated by the Ministry for Social Affairs and Health, among other experts, has suggested the establishment of a single national evaluation body for medicines in Finland (48). Harmonizing the assessment, appraisal, and decision-making activities of inpatient and outpatient medications would also make the process more comprehensible for healthcare providers, patient representatives, and citizens.

## Limitations

There are some limitations in this study. The number of interviewees was limited due to the small number of patient organizations that have experience in providing submissions to the PPB and the COHERE. Additionally, the number of authorities working with the appraisal and reimbursement processes under the Ministry for Social Affairs and Health is limited in Finland. Despite these limitations in the interviews with patient organizations and authorities, saturation was observed, and certain themes recurred throughout the interviews. Therefore, the results of the interviews can be considered representative. However, additional research is needed to better understand the potential differences between insights from patient organizations and individual citizens, the actual content of the submissions, and the significance of the input received at the different stages of the HTA process.

## Conclusions

The interviewed patient representatives appreciate involvement opportunities from the PPB and COHERE, but the impact of submissions is unclear. Authorities value insights provided by patient organizations but acknowledge their limited impact on decisions. Both patient organizations and authorities face resource constraints, emphasizing the need for clear guidelines and constructive feedback on submissions. A suggested improvement involves integrating patient representation in PPB’s and COHERE’s expert groups or decision-making bodies, drawing inspiration from established models for enhanced transparency and legitimacy (11;12;13;17;40).

## Supporting information

Tran Minh et al. supplementary material 1 Tran Minh et al. supplementary material

Tran Minh et al. supplementary material 2 Tran Minh et al. supplementary material

## Supplementary material

The supplementary material for this article can be found at http://doi.org/10.1017/S0266462324000229.
