# Supplementary material for: Experiences of patient organizations’ involvement in medicine appraisal and reimbursement processes in Finland – a qualitative study
Source: Int J Technol Assess Health Care. 2024 Jul 2;40(1):e26. doi: 10.1017/S0266462324000229 (PMC11569905; doi:10.1017/S0266462324000229)
Supplement: Tran Minh et al. supplementary material 1 — Tran Minh et al. supplementary material [file S0266462324000229sup001.pdf]

A qualitative study on experiences of patient organizations' involvement in medicine appraisal and reimbursement processes in Finland

Supplementary file 1. Interview guides

Interview guide for patient organizations*

* This translation from Finnish is intended for publication purposes and might not represent a validated translation. This study focuses on the questions that are presented in *italics*.

Processes of the Pharmaceutical Pricing Board (PPB) and the Council for Choices in Health Care in Finland (COHERE) and collaboration with the authorities

- How familiar are you with the operations of the Pharmaceutical Pricing Board (PPB) and the Council for Choices in Healthcare in Finland (COHERE)?
- Has your organization provided submissions to PPB or commented on the draft recommendations of the COHERE?
- Who makes the submissions in your organization, and how are the members of your organization involved in preparing the submissions?
- *What has been your experience of providing submissions and/or collaborating with the PPB and COHERE?*

Experiences of involvement and significance of the submissions

- *How is the involvement of patient organizations implemented in the official processes related to the reimbursement of medicines (PPB) and the preparation of medicine-related recommendations (COHERE)?*
- *Do you see that the submissions you have given have been taken into account in the PPB's / COHERE's decision making?*

Access to information

- What is your overall perception of the level of awareness of the medicines assessment, appraisal and reimbursement processes among patient organizations?
- From which actors do you obtain information on current medical treatments being assessed at the PPB / COHERE?
- Is the guidance and information provided by the authorities understandable, adequate, and up to date?
- How could the information provided by the authorities to patient organizations be improved?
- *How is information on submissions returned to the patient organization?*

Possible needs for training and support

- Do you or your organization need any information, support or training in providing submissions to PPB or commenting the draft recommendations of COHERE?
- If yes, what kind of information, support or training do you feel you need?

Interview guide for authorities**

** This translation from Finnish is intended for publication purposes and might not represent a validated translation. This study focuses on the questions that are presented in *italics*.

Collaboration with patient organizations and their involvement in the processes of the Pharmaceutical Pricing Board (PPB) and the Council for Choices in Healthcare in Finland (COHERE)

- What kind of collaboration have you had with patient organizations?
- *What is your experience of the involvement of patient organizations in the processes of PPB or COHERE?*

The role of patient organizations and the significance of their submissions

- *What role do you see for patient organizations and patient organization submissions in the operations of PPB or COHERE?*
- *How are patient organization submissions taken into account in the PPB's or the COHERE's decision making?*
- *Are the submissions made so that they are useful in the PPBs or COHERE's operations? How could they be developed in terms of content?*

Communication and possible needs for training

- How do you see the communication towards or from patient organizations working? How could it be improved?
- Have you found that patient organizations would benefit from training on preparing submissions or participating in the processes of PPB or COHERE?
- If yes, what kind of training?
