# Supplementary material for: Experiences of patient organizations’ involvement in medicine appraisal and reimbursement processes in Finland – a qualitative study
Source: Int J Technol Assess Health Care. 2024 Jul 2;40(1):e26. doi: 10.1017/S0266462324000229 (PMC11569905; doi:10.1017/S0266462324000229)
Supplement: Tran Minh et al. supplementary material 2 — Tran Minh et al. supplementary material [file S0266462324000229sup002.pdf]

**A qualitative study on experiences of patient organizations' involvement in medicine appraisal and reimbursement processes in Finland**

**Supplementary file 2.**

**Table S1. Interviews**

| Interview code | Time | Number of interviewees | Duration |
|---|---|---|---|
| PO1* | June 2021 | 1 | 43:21 |
| PO2 | June 2021 | 2 | 48:17 |
| PO3 | June 2021 | 1 | 28:03 |
| PO4 | June 2021 | 2 | 45:53 |
| PO5 | August 2021 | 1 | 28:09 |
| PO6 | August 2021 | 1 | 23:10 |
| PO7 | August 2021 | 1 | 19:46 |
| PO8 | August 2021 | 2 | 47:57 |
| PO9 | August 2021 | 2 | 46:59 |
| PO10 | October 2021 | 1 | 51:24 |
| GO1** | September 2021 | 1 | 23:09 |
| GO2 | October 2021 | 1 | 41:59 |
| GO3 | October 2021 | 1 | 55:32 |
| GO4 | October 2021 | 1 | 38:21 |
| GO5 | October 2021 | 1 | 1:05:15 |
| GO6 | November 2021 | 1 | 36:29 |
| GO7 | November 2021 | 1 | 47:12 |
| | | | |
| Total | 17 interviews | 21 persons | 681 minutes |

*PO=Patient representative(s)
**GO=Government official
